# Plasma 25-hydroxyvitamin D level and the risk of frailty among Chinese community-based oldest-old: evidence from the CLHLS study

**DOI:** 10.1186/s12877-020-01523-w

**Published:** 2020-04-06

**Authors:** Qi Xiao, Meiliyang Wu, Jinrui Cui, Mengmei Yuan, Ye Chen, Tieying Zeng

**Affiliations:** Tongji Hospital, Tongji Medical College, Huazhong University of Science and Technology, Wuhan, China

**Keywords:** China, Oldest-old, Frailty, 25(OH) D level, Biomarkers

## Abstract

**Background:**

Vitamin D deficiency has been linked to the risk of frailty. However, there are limited methods for evaluations of the potential association of vitamin D with frailty in a longevous (80+) population. The aim of this study was to examine the association between plasma 25-hydroxyvitamin D [25(OH)D] levels and the risk of frailty among the Chinese community based oldest-old.

**Methods:**

Secondary analysis of data compiled in the 2011 wave of the Chinese Longitudinal Healthy Longevity Survey (*n* = 1324) was performed. Frailty was assessed by the Study of Osteoporotic Fractures (SOF) index. Multivariate logistic regression and spline smoothing with threshold effect analysis were performed to investigate the association between 25(OH) D level and the risk of frailty after adjusting for socio-demographic variables, health characteristics and confounding biomarkers.

**Results:**

The mean age was 92.89 ± 7.92 years, and 844 (63.7%) participants were women. In all, data from 426 (33.2, 95% confidence interval, CI: 29.66–34.69) frail participants were recorded. After adjustment for confounding covariates, the level of 25(OH) D was significantly related to frailty. By spline smoothing with threshold effect analysis, a monotonically negative association between 25(OH) D and frailty was identified. Subgroup analyses revealed that the association did not differ by sex or age.

**Conclusions:**

The 25(OH) D level was inversely associated with the risk of frailty among the Chinese community-based oldest-old.

## Background

Frailty, as a geriatric syndrome, represents a reduced ability to rebuild homeostasis in response to external stressors during daily life [1]. Frailty in the elderly is well established to be related to long-term adverse health outcomes (such as falls, depression, disability, dependency, and mortality) that cannot be completely explained by ageing, functional decline, or comorbidities [2–5].

Of the numerous existing frailty measures, many often rely on measuring physical function, with some being less likely to be readily available in clinical settings and, therefore, having limited use [6–8]. Common approaches such as the Frailty Index [9, 10] and the Frailty Phenotype [11] are no exceptions. In contrast, the Study of Osteoporotic Fractures (SOF) frailty index employs only 3 simple self-reported frailty components, muscle strength, low energy, and unintentional weight loss [7]. Frailty identified through this measure has been associated with falls, disability, fractures, and death [6, 12], and the SOF index is regarded as a useful tool for assessments of the physical aspects of frailty at the population level [7, 8, 13].

Vitamin D, which is primarily synthesized in the skin upon exposure to sunlight, is necessary for human musculoskeletal health maintenance [14]; its deficiency is proven to be the cause of muscle weakness [15], sarcopenia [16], falls [17], and fracture [18]. A growing body of evidence has suggested that a low level of its active form, namely, 25-hydroxyvitamin D [25(OH)D], is related to the risk of frailty [19–33]. The underlying pathogenic mechanisms of this relationship could be explained through 3 pathways: the first is the invalidation of regulatory effects of vitamin D on calcium flux, mineral homeostasis and protein anabolism in muscle tissue [23, 24]; the second is bone metabolic disturbance by secondary hyperparathyroidism [25]; and the last is the possible anti-inflammatory property of vitamin D [26].

However, since the cutaneous synthesis of vitamin D shows great variability across populations [34], studies exploring its association in elderly Asian populations are limited. Moreover, as this cutaneous synthesis process decreases with age [35], and limited outdoor activities may also accelerate vitamin D deficiency [14], the relationship between 25(OH) D and frailty in the oldest-old people (aged ≥80 years) remains unclear. Since the oldest-old will be the fastest-growing group between now and 2050 [11], the association between 25(OH) D level and the risk of frailty in this age group may have important public health implications for health-care planning and practice.

Therefore, this study aimed to examine the association between 25(OH) D level and the risk of frailty among 1324 oldest-old adults of the eight “longevity areas” in the Chinese Longitudinal Healthy Longevity Survey (CLHLS) [36, 37]. Given the evidence in previous studies, we hypothesized that a lower level of 25(OH) D would be associated with the risk of frailty in Chinese community-based oldest-old people.

## Methods

### Study design and participants

Participants in the biomarker substudy from the 6th (2011) wave of the CLHLS were recruited in this secondary analysis. The CLHLS is the first and largest nationwide, community-based, longitudinal prospective cohort survey concerning older adults in China [38]. It provides information on the health status, socio-economic characteristics, and lifestyles of elderly individuals, including a large percentage of the oldest population [37]. The in-depth study was launched in eight “longevity areas” of China (Laizhou of Shandong Province, Xiayi of Henan Province, Zhongxiang of Hubei Province, Mayang of Hunan Province, Sanshui of Guangdong Province, Yongfu of Guangxi Autonomous Region, Chengmai of Hainan Province, Rudong of Jiangsu Province), where the density of centenarians was exceptionally high and the environmental quality was very good, as evaluated and officially designated by the expert committee of the Chinese Gerontology Association [36].

During the in-depth study, the Chinese Center for Disease Control and Prevention (CDC) local network medical doctors conducted physical examinations of the participants and collected biomarker datasets containing approximately 30 indicators in routine blood tests, blood biochemical tests, and urine tests [36]. More detailed descriptions have been previously published elsewhere [39–41].

Initially, a total of 2439 elderly participants were included in the study. We excluded those of younger age (less than 80, *n* = 834, 34.2%) and those with missing data on SOF index components (*n* = 281, 11.5%). Finally, we retained 1324 older adults in this study.

### Outcome

Consistent with previous studies of secondary analyses involving CLHLS data [42], frailty was defined by the SOF index in the current study. Three components were included in the index: underweight (defined as body mass index < 18.5), low energy level (indicated by a positive response to the question “Over the last 6 months, have you been limited in activities because of a health problem?”), and muscle strength (inability to stand up from a chair without the assistance of arms). As suggested, participants with two or more of the three components were defined as frail.

### Exposure

Fasting venous blood was collected after an overnight fast from all willing participants. Procedures for the collection and shipment of blood samples were described in detail elsewhere [14]. 25(OH) D was assayed by an enzyme-linked immunoassay using Immunodiagnostic Systems Limited (IDS Ltd., Boldon, UK). The 25(OH) D level was expressed as nmol/L.

### Covariates

We adjusted for socio-demographic variables, health characteristics and confounding biomarkers in the models. Socio-demographic variables included age, sex (female/male), marital status (married/other), residence (rural/other), education level (no schooling/≥1 year of schooling), and co-residence [with family member(s)/other].

Health characteristics included lifestyles and chronic diseases. Lifestyles consisted of smoking (yes/no), drinking (yes/no), and regular exercise (yes/no) at present. Chronic diseases included hypertension (yes/no), diabetes mellitus (yes/no), heart diseases (yes/no), cerebrovascular diseases (yes/no), and respiratory diseases (yes/no). Hypertension was defined as systolic blood pressure ≥ 140 mmHg and/or diastolic blood pressure ≥ 90 mmHg [43]. Diabetes mellitus was diagnosed by fasting plasma glucose≥7.0 mmol/L [14, 44]. Other diseases were identified by self-report.

Confounding biomarkers were 11 indicators on routine blood tests and blood biochemistry tests [36]. According to previous relevant studies [19], these 11 indicators, which were largely investigated in relation to frailty, were analysed in this study: 1) inflammatory marker: C reactive protein (CRP); 2) immune marker: counts of leukocytes (WBC); 3) clinical markers: plasma albumin (ALB), total cholesterol (CHO), serum creatinine (CREA), high-density lipoprotein cholesterol (HDLC), low-density lipoprotein cholesterol (LDLC), triglyceride (TG), and haemoglobin (HGB); and 4) oxidative stress markers: malondialdehyde (MDA) and superoxide dismutase (SOD). All standard laboratory techniques were performed by the central clinical laboratory at Capital Medical University in Beijing.

Overall, few data points for most confounding variables were missing (1.05%). For the missing values, we performed multiple imputations by chained equations to increase the predictive power [45]. The distributions of the observed data and imputed data are described in **Supplementary Table S1 (see** Additional file 1**)**. For all the covariates, the distributions of observed and imputed values were similar.

### Statistical analysis

Categorical variables were expressed as numbers and percentages, and continuous data were described as the mean (standard deviation, SD) or median (interquartile range, IQR). Characteristics among groups were compared by ANOVA, Kruskal–Wallis test or χ^2^ test. The IQR of the 25(OH) D level was used to divide the data into four categories. The cutoff points were 26.13, 35.89, and 50.00 nmol/L.

We used multilayer logistic regression models based on the likelihood ratio test (LRT) to determine the association between 25(OH) D level and the risk of frailty. The Box-Tidwell method was used to test the linearity between logit P and all continuous variables [46]. Therefore, we used continuous terms for all the confounding biomarkers and categorized age as subgroups with 80–89, 90–99, and ≥ 100 years. Data are reported as odds ratios (ORs) and 95% confidence intervals (CIs) in both unadjusted and adjusted logistic regression models. A *p*-value of the Hosmer-Lemeshow test > 0.05 indicated reasonable goodness of fit [47].

Different from previous studies, to examine the linear trend across levels of 25(OH) D, we further performed spline smoothing analysis and threshold effect analysis in the current study, which were relatively novel in studies examining the respondents’ dose-response relationship between 25(OH) D and frailty. Instead of a priori assumptions, spline smoothing analysis is a form of mixed modelling based on the generalized additive model (GAM) [48], whereby a set of associated items, for example, 25(OH) D and frailty, can visually demonstrate the linear or curvilinear relationship by figures. The threshold effect analysis, which is based on the piece-wise regression model [49], can further examine whether this relationship is segmental.

Subgroup analyses and their interactions were tested to explore whether sex and age subgroups would confound the association between 25(OH) D level and frailly. Sensitivity analysis was performed in participants with complete variables and multiple imputations separately.

A two-tailed *p*-value < 0.05 was considered statistically significant in all analyses. Statistical analyses were conducted by IBM SPSS Statistics Version 22.0, except that the spline smoothing analysis, threshold effect analysis, and multiple imputations were performed by R software Version 3.4.3 (http://www.R-project.org) and Empower® (www.empowerstats.com).

## Results

### Sample characteristics

The characteristics of the participants were compared according to the 25(OH) D level categories. The full detailed characteristics of all participants are shown in Table 1. The mean ± SD age of the study population was 92.89 ± 7.92 years, and 63.7% were women (*n* = 844). The number of participants with frailty was 426 (33.2, 95% CI: 29.66–34.69). The median 25(OH) D concentration was 35.89 nmol/L, and participants with higher 25(OH) D levels (35.89–50.00, > 50.00 nmol/L) were significantly younger than those with lower levels (≤26.13, 26.13–35.89 nmol/L) and were more likely to be male, married, have ≥1 year of schooling and perform regular exercise.

**Table 1:** Participant characteristics

Variables	All participants (*n* = 1324)	Categories (nmol/L)				Statistics ^a^
		Q_1_ (≤26.13)	Q_2_ (26.13–35.89)	Q_3_ (35.89–50.00)	Q_4_ (> 50.00)	
**Socio-demographics**, *n* (%)						
Age (80–112), M (SD)	92.89 (7.92)	95.63 (7.49)	93.37 (7.72)	91.85 (7.85)	90.70 (7.78)	25.207^***^
Female	844 (63.7)	251 (75.8)	236 (71.3)	200 (60.2)	157 (47.6)	68.190^***^
Married	294 (22.3)	44 (13.3)	60 (18.2)	83 (25.1)	107 (32.7)	40.503^***^
Rural	1124 (84.9)	282 (85.2)	282 (85.2)	265 (79.8)	295 (89.4)	11.925^**^
No schooling	998 (76.4)	279 (85.8)	257 (78.4)	242 (74.2)	220 (67.1)	33.410^***^
With household member(s)	950 (73.2)	263 (82.2)	236 (72.2)	225 (69.7)	226 (69.1)	25.873^***^
**Health characteristics**, *n* (%)						
Smoking	148 (11.3)	28 (8.5)	39 (11.8)	37 (11.2)	44 (13.5)	4.337
Drinking	167 (12.7)	32 (9.7)	41 (12.5)	45 (13.6)	49 (14.9)	0.218
Regular exercise	178 (13.9)	27 (8.4)	38 (11.9)	54 (16.7)	59 (18.6)	17.317^***^
Hypertension	785 (62.2)	197 (61.9)	195 (62.1)	192 (61.5)	201 (63.0)	0.155
Diabetes mellitus	98 (7.4)	28 (8.5)	25 (7.6)	24 (7.3)	21 (6.4)	1.057
Heart diseases	91 (7.0)	24 (7.4)	24 (7.4)	27 (8.4)	16 (4.9)	3.398
Cerebrovascular diseases	102 (7.8)	34 (10.4)	31 (9.5)	18 (5.5)	19 (5.8)	8.630^*^
Respiratory diseases	116 (8.9)	29 (9.0)	32 (9.8)	23 (7.0)	32 (9.8)	2.080
**Biomarkers**, M (IQR)						
CRP (mg/L)	1.01 (0.41,2.93)	1.12 (0.38,3.35)	0.93 (0.43,3.05)	0.96 (0.41,2.54)	1.09 (0.39,2.75)	1.491
ALB (g/L)	39.10 (35.90,42.40)	37.90 (35.30,41.40)	38.60 (35.48,42.12)	39.70 (36.70,42.93)	39.90 (37.20,42.80)	29.923^***^
CHO (mmol/L)	4.16 (3.52,4.79)	4.03 (3.49,4.72)	4.21 (3.51,4.79)	4.21 (3.47,4.97)	4.20 (3.70,4.78)	4.186
CREA (mmol/L)	78 (65,96)	69 (60,85)	77 (63,93)	82 (69,98)	87 (71,102)	76.765^***^
HDLC (mmol/L)	1.23 (1.03,1.49)	1.20 (1.01,1.45)	1.25 (1.04,1.51)	1.27 (1.03,1.55)	1.23 (1.04,1.46)	5.065
LDLC (mmol/L)	2.45 (1.94,3.02)	2.40 (1.92,2.97)	2.42 (1.89,3.05)	2.41 (1.86,3.08)	2.54 (2.04,3.00)	5.147
TG (mmol/L)	0.79 (0.59,1.10)	0.78 (0.59,1.07)	0.79 (0.58,1.09)	0.82 (0.61,1.16)	0.77 (0.57,1.07)	6.429
SOD (IU/mL)	58.53 (53.43,63.24)	56.75 (51.75,62.97)	58.18 (53.49,63.20)	58.75 (53.33,63.06)	59.39 (55.39,64.24)	18.975^***^
MDA (μmol/L)	4.71 (3.73,5.79)	4.81 (3.93,5.89)	4.87 (3.88,5.91)	4.84 (3.82,5.83)	4.33 (3.25,5.55)	27.303^***^
WBC (10^9^/L)	5.30 (4.30,6.40)	4.80 (4.00,6.00)	5.10 (4.10,6.10)	5.60 (4.57,6.60)	5.60 (5.60,6.80)	34.983^***^
HGB (g/L)	118 (106,131)	121 (110,133)	120 (107,132)	116 (105,129)	117 (105,131)	11.618^***^
**Frailty**, *n* (%)	426 (33.2)	162 (48.9)	112 (33.8)	93 (28.0)	59 (17.9)	76.606^***^

*M (SD)* mean (standard deviation), *M (IQR)* median (interquartile range)

^a^ Coefficient of ANOVA, Kruskal–Wallis test or χ^2^ test among categories of plasma 25(OH) D level

^*^ < 0.05, ^**^ < 0.01, ^***^ < 0.001

Abbreviations: *CRP* C reactive protein, *ALB* plasma albumin, *CHO* total cholesterol, *CREA* plasma creatine, *HDLC* high-density lipoprotein cholesterol, *LDLC* low-density lipoprotein cholesterol, *SOD* superoxide dismutase, *TG* triglyceride, *SOD* superoxide dismutase, *MDA* malondialdehyde, *WBC* white blood cell count, *HGB* haemoglobin

### Association between the level of 25(OH) D and the risk of frailty

As shown in Table 1, 48.9%, 33.8%, 28.0% and 17.9% of participants in the lowest to highest 25(OH) D categories reported frailty. There was a significant inverse association between categorical 25(OH) D level and the risk of frailty in the multivariate logistic regression models. The ORs and 95% CIs for the association between categories of 25(OH) D level and frailty are presented in Table 2. After eliminating the interferences of all confounding factors, the ORs of frailty were 3.239 (95% CI: 2.113–4.967, *p* < 0.001) for the lowest category (≤26.13 nmol/L) of 25(OH) D level, 2.341 (95% CI: 1.519–3.609, *p* < 0.001) for the second-lowest category (26.13–35.89 nmol/L), and 1.703 (95% CI: 1.088–2.664, *p* = 0.20) for the third-lowest category (30.33–44.46 nmol/L) compared to the highest level subgroup (> 50.00 nmol/L).

**Table 2:** The associations between serum level of 25(OH) D (nmol/L) and the risk of frailty

Variables	Model 1 ^a^	Model 2 ^b^	Model 3 ^c^	Model 4 ^d, e^
Categories				
≤ 26.13	4.964 (3.332,7.396) ^***^	3.472 (2.273,5.303) ^***^	3.437 (2.248,5.255) ^***^	3.239 (2.113,4.967) ^***^
26.13–35.89	2.822 (1.881,4.234) ^***^	2.414 (1.571,3.710) ^***^	2.420 (1.573,3.723) ^***^	2.341 (1.519,3.609) ^***^
35.89–50.00	1.835 (1.204,2.797) ^**^	1.526 (1.102, 2.683) ^*^	1.722 (1.102,2.692) ^*^	1.703 (1.088,2.664) ^*^
> 50.00	reference	reference	reference	reference

^a^ Unadjusted model, OR (95% CI)

^b^ Adjusted for **socio-demographics** (age, sex, marital status, residence, education level, and co-residence), OR (95% CI)

^c^ Adjusted for **socio-demographics** (age, sex, marital status, residence, education level, and co-residence) and **health characteristics** (smoking, drinking, regular exercise, hypertension, diabetes mellitus, heart diseases, cerebrovascular diseases, and respiratory diseases), OR (95% CI)

^d^ Adjusted for **socio-demographics** (age, sex, marital status, residence, education level, and co-residence), **health characteristics** (smoking, drinking, regular exercise, hypertension, diabetes mellitus, heart diseases, cerebrovascular diseases, and respiratory diseases) and **confounding biomarkers** (CRP, ALB, CHO, CREA, HDLC, LDLC, TG, SOD, MDA, WBC, and HGB), OR (95% CI)

^e^*p*-value for the Hosmer-Lemeshow test was 0.653; prediction in accuracy was 74.3% in model 4

^*^ < 0.05, ^**^ < 0.01, ^***^ < 0.001

### The dose-response relationship between the level of 25(OH) D and the risk of frailty

**Fig. 1:**
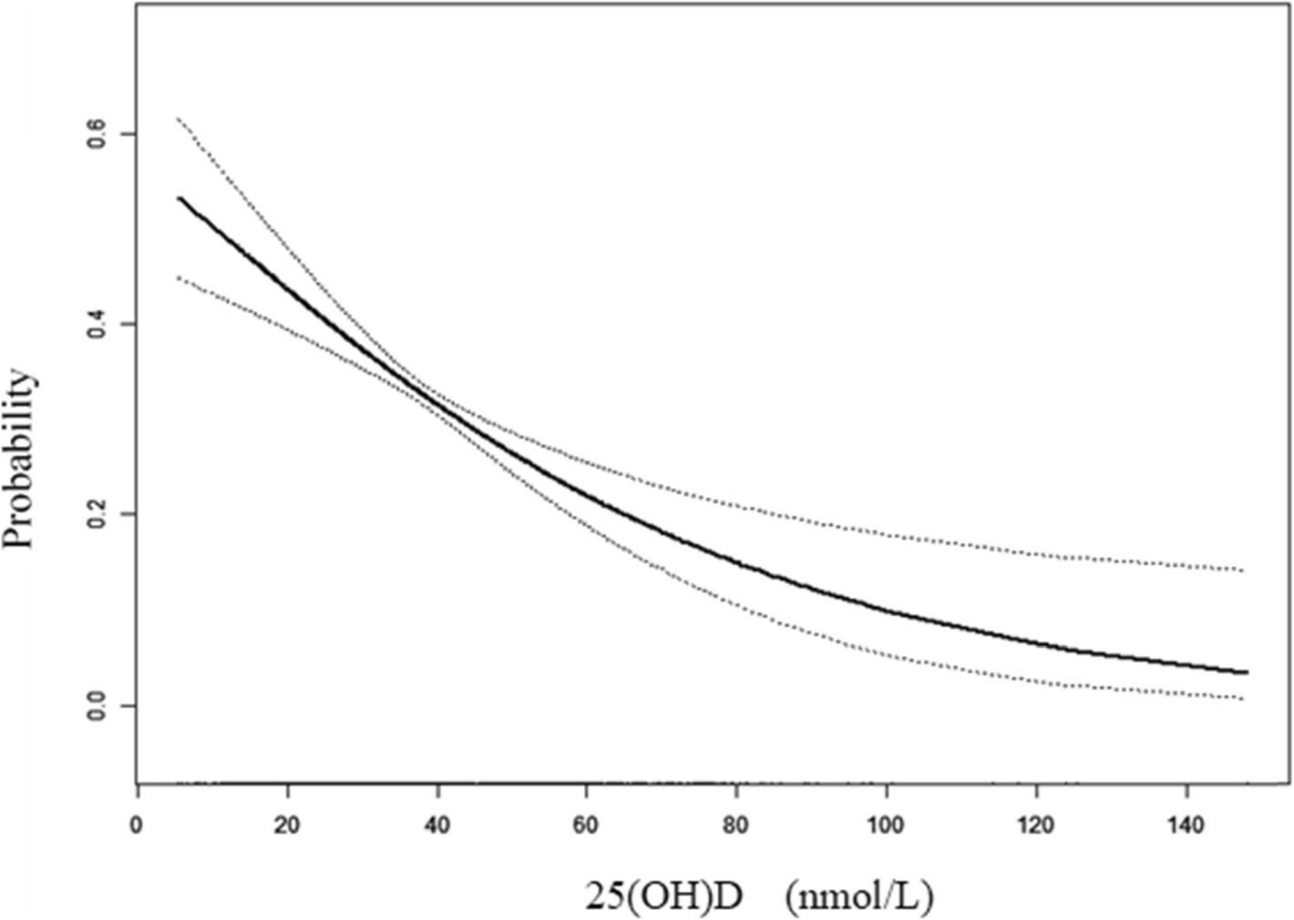
The dose-response relationship of 25(OH) D level and the risk of frailty adjusted for socio-demographics (age, sex, marital status, residence, education level, and co-residence), health characteristics (smoking, drinking, regular exercise, hypertension, diabetes mellitus, heart diseases, cerebrovascular diseases, and respiratory diseases) and confounding biomarkers (CRP, ALB, CHO, CREA, HDLC, LDLC, TG, SOD, MDA, WBC, and HGB). Lines = estimated probability of frailty with 25(OH) D, dotted lines = 95% confidence intervals

Consistent with the results displayed in Table 2, a continuous negative curve was observed between 25(OH) D and the risk of frailty in the analysis using spline smoothing (*p* for trend < 0.001, Fig. 1). Table 3 shows that the *p*-value of the log-likelihood ratio test in the threshold effect analysis was 0.317 in the adjusted model, which showed that the tendency of the association between 25(OH) D and frailty was monotonical with no inflection.

**Table 3:** Threshold effect analysis of 25(OH) D (nmol/L) using the piece-wise regression model

Variables	Crude ^a^ OR (95% CI)	Adjusted ^b^ OR (95% CI)
Continuous	0.967 (0.960, 0.975) ^***^	0.975 (0.965, 0.984) ^***^
Cutoff		
≤ 33.96	0.948 (0.929, 0.967) ^***^	0.963 (0.939, 0.988) ^***^
> 33.96	0.978 (0.966, 0.989) ^***^	0.981 (0.966, 0.996) ^*^
*p*-value of log-likelihood ratio test	0.032	0.317

^a^ Crude: no adjustment

^b^ Adjusted for **socio-demographics** (age, sex, marital status, residence, education level, and co-residence), **health characteristics** (smoking, drinking, and regular exercise, hypertension, diabetes mellitus, heart diseases, cerebrovascular diseases, and respiratory diseases) and **confounding biomarkers** (CRP, ALB, CHO, CREA, HDLC, LDLC, TG, SOD, MDA, WBC, and HGB)

^*^ < 0.05, ^**^ < 0.01, ^***^ < 0.001

### Subgroup analyses

**Fig. 2:**
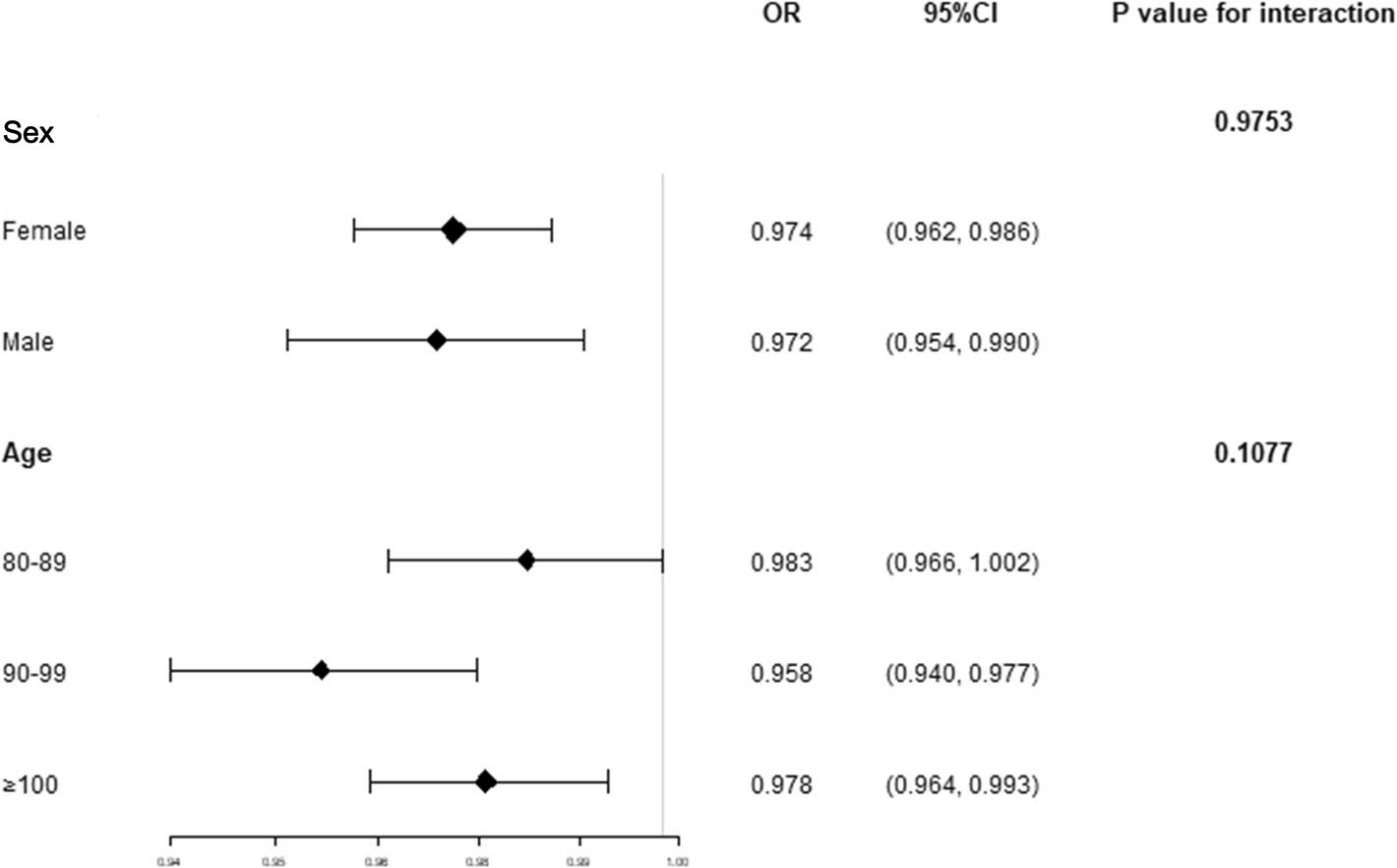
Subgroup analyses for the association between 25(OH) D and frailty adjusted for marital status, residence, education level, and co-residence; smoking, drinking, and regular exercise; hypertension, diabetes mellitus, heart diseases, cerebrovascular diseases, and respiratory diseases; and CRP, ALB, CHO, CREA, HDLC, LDLC, TG, SOD, MDA, WBC, and HGB

Subgroup analyses showed that the *p*-value for interaction was 0.9753 for sex and 0.1077 for age, which revealed that the association of 25(OH) D level with frailty did not significantly differ by sex or age after adjusting for a series of covariates (Fig. 2).

### Sensitivity analysis

We performed the multivariate analysis in those participants with complete variables and multiple imputations separately. As displayed in **Supplementary Table S2 (see** Additional file 1**)**, the present findings showed similar results.

## Discussion

In this community-based study, after adjustment for a variety of potential confounding factors, we observed a robust and monotonically negative association of 25(OH) D with frailty among a group of oldest-old individuals in China. In addition, by examining the relationship between different subgroups of participants, we also indicated that this association was consistent across sex and age groups.

### Comparison with other studies

The relationship between 25(OH) D level and frailty has rarely been investigated among the oldest-old individuals. As hypothesized, the present findings suggested that the 25(OH) D level was related to frailty after adjustment for numerous confounders. The findings from our study were relatively consistent with those of previous studies involving older adults in the Netherlands [25, 32], Mexico [23], northern Taiwan [33] and Germany [31].

Limited studies have demonstrated the shape of the association between 25(OH) D level and frailty. In a study of 1606 old men aged 73.8 ± 5.9 years in the USA, a segmented negative curvilinear association between 25(OH) D level and odds of frailty was identified [21]. However, this association was not observed among 6307 old women in America [20] and was replaced by a U-shaped curvilinear association. For the present study, we identified a robust and monotonically negative association between 25(OH) D and frailty in older Chinese adults.

Chronic diseases, lifestyles, and some biomarkers have been studied as potential risk factors of frailty in the existing literature [11, 19, 24]. However, the association between 25(OH) D and frailty was not substantially confounded after adding these covariates in the models of our study. This suggested that 25(OH) D was independently associated with the risk of frailty. However, it remains inconclusive whether other possible factors not included in this study might contribute to the relationship between 25(OH) D and frailty.

Due to differences in latitudes, seasons, measurements of frailty, the adequacy of adjustment for potential confounders, and clinical heterogeneity between races and ethnicities [34], some variations from previous studies were also found in this study. First, our study reported that the median 25(OH) D level of elderly individuals was 35.89 nmol/L, which was lower than the median levels of those participants in Taiwan [33], the Netherlands [25], and Germany [27]. In addition, the prevalence of frailty was 33.2%, which was much higher than the 13% reported in Japanese elderly [8] and the 17% reported in US older women [6]. Since the 25(OH) D level and frailty status are closely related to ageing [19], these differences could also be partly explained by the oversampling of oldest-old people in this study.

Second, the association between 25(OH) D level and frailty has been reported to be different across sex [20, 21, 27, 28, 30]. A study of participants in Italy identified that vitamin D insufficiency was associated with frailty only in men [30]. However, conflicting results were reported in studies involving older women in the USA [24], Spain [29], and Portugal [28]. In this regard, our study detected that 25(OH) D level was associated with frailty regardless of sex, which was similar to the findings of the study of individuals in Germany [27]. In addition, our study also indicated that this association did not differ among octogenarian, nonagenarian, and centenarian subgroups.

### Strength and limitations

The strength of the current study was the large number of Chinese community-based participants with the collection of plasma blood samples during the survey. This allowed us to investigate the shape of the association between 25(OH) D level and frailty and to adjust for important potential confounding variables. To our knowledge, this was the first study that investigated the relationship between 25(OH) D level and frailty in a nationwide study of Chinese oldest-old people. The present study included a large population of older Chinese adults aged 80 years and over, which allowed robust conclusions to be drawn with respect to these participants.

This study also has certain limitations. First, a dichotomous outcome measure for frailty was used in this study; hence, the pre-frail status was not taken into account. Second, some of the clinical diseases were self-reported. For this condition, we adjusted hypertension and diabetes status by clinical data in place of self-reporting to eliminate information bias. Third, this was a descriptive cross-sectional design that did not allow us to evaluate whether a change in 25(OH) D level was a cause or a consequence of frailty.

## Conclusions

With a population-based design, this study indicates that the 25(OH) D level is monotonically and negatively associated with frailty in the Chinese community-dwelling population. The results of the present study, along with those of other existing epidemiological studies, reinforce the importance of the investigation of the full explanation of the association between 25(OH) D and frailty. Further longitudinal studies are needed to verify our initial cross-sectional findings so that we may identify an effective intervention to stem the rapidly increasing prevalence of frailty associated with an ageing population.

## Supplementary information

**Additional file 1 Supplementary Table S1** Distribution of observed data and imputed data. **Supplementary Table S2** Sensitivity analysis of 25(OH) D (nmol/L) with risk of frailty.

## Data Availability

The CLHLS questionnaires are available at https://sites.duke.edu/centerforaging/ programs/chinese-longitudinal-healthy-longevity-survey-clhls/survey-documentation/ questionnaires/. The full datasets used in this analysis are available from the corresponding author upon reasonable request.

## Abbreviations

CLHLS: Chinese Longitudinal Healthy Longevity Survey
25(OH)D: 25-hydroxyvitamin D
SOF: Study of Osteoporotic Fractures
FI: Frailty Index
CGA: Comprehensive geriatric assessment
CHS: Cardiovascular Health Study
CDC: Center for Disease Control and Prevention
SD: Standard variance
IQR: Interquartile range
LRT: Likelihood Ratio Test
OR: Odds ratio
CI: Confidence interval
GAM: Generalized additive model
CRP: C reactive protein
ALB: Plasma albumin
CHO: Total cholesterol
CREA: Plasma creatine
SOD: Superoxide dismutase
HDLC: High-density lipoprotein cholesterol
LDLC: Low-density lipoprotein cholesterol
TG: Triglyceride
SOD: Superoxide dismutase
MDA: Malondialdehyde
WBC: White blood cell count
HGB: Hemoglobin

## References

[1] Clegg A, Young J, Iliffe S, Rikkert MO, Rockwood K (2013). Frailty in elderly people. Lancet.

[2] Collard RM, Boter H, Schoevers RA, Oude Voshaar RC (2012). Prevalence of frailty in community-dwelling older persons: a systematic review. J Am Geriatr Soc.

[3] Kojima G (2015). Frailty as a predictor of future falls among community-dwelling older people: a systematic review and meta-analysis. J Am Med Dir Assoc.

[4] Kojima G (2017). Frailty as a predictor of disabilities among community-dwelling older people: a systematic review and meta-analysis. Disabil Rehabil.

[5] At J, Bryce R, Prina M, Acosta D, Ferri CP, Guerra M (2015). Frailty and the prediction of dependence and mortality in low- and middle-income countries: a 10/66 population-based cohort study. BMC Med.

[6] Ensrud KE, Ewing SK, Taylor BC, Fink HA, Cawthon PM, Stone KL (2008). Comparison of 2 frailty indexes for prediction of falls, disability, fractures, and death in older women. Arch Intern Med.

[7] Erusalimsky JD, Grillari J, Grune T, Jansen-Duerr P, Lippi G, Sinclair AJ (2016). In search of 'Omics'-based biomarkers to predict risk of frailty and its consequences in older individuals: the FRAILOMIC initiative. Gerontology..

[8] Cable N, Hiyoshi A, Kondo N, Aida J, Sjoqvist H, Kondo K. Identifying frail-related biomarkers among community-dwelling older adults in Japan: a research example from the Japanese Gerontological evaluation study. Biomed Res Int. 2018;8:5362948. 10.1155/2018/5362948 PMC5828560 29607322

[9] Rockwood K, Mitnitski A (2007). Frailty in relation to the accumulation of deficits. J Gerontol A Biol Sci Med Sci.

[10] Rockwood K, Mitnitski A (2011). Frailty defined by deficit accumulation and geriatric medicine defined by frailty. Clin Geriatr Med.

[11] Fried LP, Tangen CM, Walston J, Newman AB, Hirsch C, Gottdiener J (2001). Frailty in older adults: evidence for a phenotype. J Gerontol A Biol Sci Med Sci.

[12] Bradlee ML, Mustafa J, Singer MR, Moore LL (2017). High-protein foods and physical activity protect against age-related muscle loss and functional decline. J Gerontol A Biol Sci Med Sci.

[13] Buta BJ, Walston JD, Godino JG, Park M, Kalyani RR, Xue Q-L (2016). Frailty assessment instruments: systematic characterization of the uses and contexts of highly-cited instruments. Ageing Res Rev.

[14] Matchar DB, Chei CL, Yin ZX, Koh V, Chakraborty B, Shi XM (2016). Vitamin D levels and the risk of cognitive decline in Chinese elderly people: the Chinese longitudinal healthy longevity survey. J Gerontol A Biol Sci Med Sci.

[15] Bischoff-Ferrari HA, Dietrich T, Orav EJ, Hu FB, Zhang Y, Karlson EW (2004). Higher 25-hydroxyvitamin D concentrations are associated with better lower-extremity function in both active and inactive persons aged > or =60 y. Am J Clin Nutr.

[16] Visser M, Deeg DJ, Lips P (2003). Low vitamin D and high parathyroid hormone levels as determinants of loss of muscle strength and muscle mass (sarcopenia): the longitudinal aging study Amsterdam. J Clin Endocrinol Metab.

[17] Murad MH, Elamin KB, Abu Elnour NO, Elamin MB, Alkatib AA, Fatourechi MM (2011). Clinical review: the effect of vitamin D on falls: a systematic review and meta-analysis. J Clin Endocrinol Metab.

[18] Hill TR, Aspray TJ (2017). The role of vitamin D in maintaining bone health in older people. Ther Adv Musculoskelet Dis.

[19] Kane AE, Sinclair DA (2019). Frailty biomarkers in humans and rodents: current approaches and future advances. Mech Ageing Dev.

[20] Ensrud KE, Ewing SK, Fredman L, Hochberg MC, Cauley JA, Hillier TA (2010). Circulating 25-hydroxyvitamin D levels and frailty status in older women. J Clin Endocrinol Metab.

[21] Ensrud KE, Blackwell TL, Cauley JA, Cummings SR, Barrett-Connor E, Dam TT (2011). Circulating 25-hydroxyvitamin D levels and frailty in older men: the osteoporotic fractures in men study. J Am Geriatr Soc.

[22] Wong YY, McCaul KA, Yeap BB, Hankey GJ, Flicker L (2013). Low vitamin D status is an independent predictor of increased frailty and all-cause mortality in older men: the health in men study. J Clin Endocrinol Metab.

[23] Gutierrez-Robledo LM, Avila-Funes JA, Amieva H, Meillon C, Acosta JL, Navarrete-Reyes AP (2016). Association of low serum 25-hydroxyvitamin D levels with the frailty syndrome in Mexican community-dwelling elderly. Aging Male.

[24] Buta B, Choudhury PP, Xue QL, Chaves P, Bandeen-Roche K, Shardell M (2017). The Association of Vitamin D Deficiency and Incident Frailty in older women: the role of Cardiometabolic diseases. J Am Geriatr Soc.

[25] Vaes AMM, Brouwer-Brolsma EM, Toussaint N, de Regt M, Tieland M, van Loon LJC (2019). The association between 25-hydroxyvitamin D concentration, physical performance and frailty status in older adults. Eur J Nutr.

[26] van den Berg KS, Arts MHL, Collard RM (2018). Vitamin D deficiency and course of frailty in a depressed older population.

[27] Spira D, Buchmann N, Konig M, Rosada A, Steinhagen-Thiessen E, Demuth I (2019). Sex-specific differences in the association of vitamin D with low lean mass and frailty: Results from the Berlin Aging Study II. Nutrition (Burbank, Los Angeles County, Calif).

[28] Sousa-Santos AR, Afonso C, Santos A (2018). The association between 25(OH) D levels, frailty status and obesity indices in older adults. PLoS One.

[29] Alvarez-Ríos AI, Guerrero JM, García-García FJ, Rodríguez-Mañas L, Medrano-Campillo P, de la Torre Lanza MA (2015). Associations between frailty and serum N-terminal propeptide of type I procollagen and 25-hydroxyvitamin D in older Spanish women: the Toledo study for healthy aging. Exp Gerontol.

[30] Shardell M, Hicks GE, Miller RR, Kritchevsky S, Andersen D, Bandinelli S (2009). Association of low Vitamin D Levels with the frailty syndrome in men and women. J Gerontol A Biol Sci Med Sci.

[31] Pabst G, Zimmermann AK, Huth C, Koenig W, Ludwig T, Zierer A (2015). Association of low 25-hydroxyvitamin D levels with the frailty syndrome in an aged population: results from the KORA-age Augsburg study. J Nutr Health Aging.

[32] Puts MTE, Visser M, Twisk JWR, Deeg DJH, Lips P (2005). Endocrine and inflammatory markers as predictors of frailty. Clin Endocrinol.

[33] Chang C-I, Chan D-C, Kuo K-N, Hsiung CA, Chen C-Y (2010). Vitamin D insufficiency and frailty syndrome in older adults living in a northern Taiwan community. Arch Gerontol Geriatr.

[34] Bahrami A, Sadeghnia HR, Tabatabaeizadeh SA, Bahrami-Taghanaki H, Behboodi N, Esmaeili H (2018). Genetic and epigenetic factors influencing vitamin D status. J Cell Physiol.

[35] MacLaughlin J, Holick MF (1985). Aging decreases the capacity of human skin to produce vitamin D3. J Clin Invest.

[36] Zeng Y, Vaupel JW (2019). Chinese Longitudinal Healthy Longevity Survey (CLHLS), Biomarkers Datasets, 2009, 2012, 2014. Inter-university Consortium for Political and Social Research [distributor].

[37] Zeng Y, Vaupel J, Xiao Z, Liu Y, Zhang C (2017). Chinese Longitudinal Healthy Longevity Survey (CLHLS), 1998–2014. Inter-university Consortium for Political and Social Research [distributor].

[38] Xiao Q, Wu M, Zeng T. Social support networks in Chinese older adults: health outcomes and health related behaviors: a path analysis. Aging Ment Health. 2019;23(10):1382-90. 10.1080/13607863.2018.1488941. 10.1080/13607863.2018.1488941 PMC8435352 30691291

[39] Zeng Y, Feng Q, Hesketh T, Christensen K, Vaupel JW (2017). Survival, disabilities in activities of daily living, and physical and cognitive functioning among the oldest-old in China: a cohort study. Lancet..

[40] Lv X, Li W, Ma Y, Chen H, Zeng Y, Yu X (2019). Cognitive decline and mortality among community-dwelling Chinese older people. BMC Med.

[41] Liu Z, Han L, Feng Q, Dupre ME, Gu D, Allore HG (2019). Are China’s oldest-old living longer with less disability? A longitudinal modeling analysis of birth cohorts born 10 years apart. BMC Med.

[42] Lv YB, Gao X, Yin ZX, Chen HS, Luo JS, Brasher MS (2018). Revisiting the association of blood pressure with mortality in oldest old people in China: community based, longitudinal prospective study. Bmj.

[43] Sun D, Sun X, Xu Y, Wu T, Tao L (2019). Superoxide dismutase activity and risk of cognitive decline in older adults: findings from the Chinese longitudinal healthy longevity survey. Exp Gerontol.

[44] Yin ZX, Shi XM, Kraus VB, Fitzgerald SM, Qian HZ, Xu JW (2012). High normal plasma triglycerides are associated with preserved cognitive function in Chinese oldest-old. Age Ageing.

[45] Dupre ME, Gu D, Warner DF, Yi Z (2009). Frailty and type of death among older adults in China: prospective cohort study. Bmj.

[46] Gaudry MJ, Wills M (1978). Estimating the functional form of travel demand models. Transp Res.

[47] Cole T, Hosmer DW, Lemeshow S (1989). Applied logistic regression.

[48] Turlach B (2016). Spline Smoothing.

[49] Onukogu IB. When is Piece-Wise Regression Really Necessary? BIOM J. 1984;26:559–66.

