# Supplementary material for: Plasma 25-hydroxyvitamin D level and the risk of frailty among Chinese community-based oldest-old: evidence from the CLHLS study
Source: BMC Geriatr. 2020 Apr 6;20:126. doi: 10.1186/s12877-020-01523-w (PMC7133005; doi:10.1186/s12877-020-01523-w)
Supplement: Supplementary file 1 — Additional file 1 Supplementary Table S1 Distribution of observed data and imputed data. Supplementary Table S2 Sensitivity analysis of 25(OH) D (nmol/L) with risk of frailty. [file 12877_2020_1523_MOESM1_ESM.docx]

Supplementary Appendix

Table of Contents

**Supplementary Table S1.** Distribution of observed data and imputed data. (pages 2-4)

**Supplementary Table S2.** Sensitivity analysis of 25(OH)D (nmol/L) with risk of frailty. (pages 5)

| **Supplementary Table S1.** Distribution of observed data and imputed data. | | | | | | | | | | |
| --- | --- | --- | --- | --- | --- | --- | --- | --- | --- | --- |
| Variables | Observed | | Imputed 1 | | Imputed 2 | Imputed 3 | | Imputed 4 | Imputed 5 | |
| **Socio-demographics** |  | |  | |  |  | |  |  | |
| Age, M (SD) | 92.89 (7.92) | | 92.89 (7.92) | | 92.89 (7.92) | 92.89 (7.92) | | 92.89 (7.92) | 92.89 (7.92) | |
| Female, n (%) | 844 (63.7) | | 844 (63.7) | | 844 (63.7) | 844 (63.7) | | 844 (63.7) | 844 (63.7) | |
| Married, n (%) | 294 (22.3) | | 295 (22.3) | | 295 (22.3) | 296 (22.4) | | 295 (22.3) | 295 (22.3) | |
| Rural, n (%) | 1124 (84.9) | | 1124 (84.9) | | 1124 (84.9) | 1124 (84.9) | | 1124 (84.9) | 1124 (84.9) | |
| No schooling, n (%) | 1013 (76.5) | | 1013 (76.5) | | 1013 (76.5) | 1011 (76.4) | | 1011 (76.4) | 1013 (76.5) | |
| With household member(s), n (%) | 950 (73.2) | | 967 (73.0) | | 964 (72.8) | 973 (73.5) | | 967 (73.0) | 969 (73.2) | |
| **Health characteristics** |  | |  | |  |  | |  |  | |
| Smoking, n (%) | 149 (11.3) | | 148 (11.2) | | 148 (11.2) | 148 (11.2) | | 148 (11.2) | 148 (11.2) | |
| Drinking, n (%) | 167 (12.6) | 167 (12.6) | | 168 (12.7) | | 168 (12.7) | 168 (12.7) | | 168 (12.7) |  |
| Regular exercise, n (%) | 178 (13.4) | | 184 (13.9) | | 181 (13.7) | 184 (13.9) | | 183 (13.8) | 181 (13.7) | |
| Hypertension, n (%) | 785 (59.3) | | 827 (62.5) | | 820 (61.9) | 823 (62.2) | | 824 (62.2) | 830 (62.7) | |
| Diabetes mellitus, n (%) | 98 (7.4) | | 98 (7.4) | | 98 (7.4) | 98 (7.4) | | 98 (7.4) | 98 (7.4) | |
| Heart diseases, n (%) | 91 (6.9) | | 93 (7.0) | | 94 (7.1) | 93 (7.0) | | 92 (6.9) | 94 (7.1) | |
| Cerebrovascular diseases, n (%) | 102 (7.7) | | 103 (7.8) | | 102 (7.7) | 107 (8.1) | | 103 (7.8) | 103 (7.8) | |
| Respiratory diseases, n (%) | 116 (8.8) | | 118 (8.9) | | 117 (8.8) | 116 (8.8) | | 118 (8.9) | 117 (8.8) | |
| **Biomarkers**, M (IQR) |  | |  | |  |  | |  |  | |
| CRP (mg/L) | 1.01 (0.41,2.93) | | 1.01 (0.41,2.93) | | 1.01 (0.41,2.93) | 1.01 (0.41,2.95) | | 1.01 (0.41,2.93) | 1.02 (0.41,2.95) | |
| ALB (g/L) | 39.10 (35.90,42.40) | | 39.10 (35.82,42.40) | | 39.10 (35.90,42.40) | 39.10 (35.90,42.40) | | 39.10 (35.90,42.40) | 39.10 (35.90,42.40) | |
| CHO (mmol/L) | 4.16 (3.52,4.79) | | 4.16 (3.52,4.79) | | 4.16 (3.52,4.80) | 4.16 (3.52,4.79) | | 4.16 (3.53,4.79) | 4.16 (3.52,4.79) | |
| CREA (mmol/L) | 78 (65,96) | | 78 (65,96) | | 78 (65,96) | 78 (65,96) | | 79 (65,96) | 78 (65,96) | |
| HDLC (mmol/L) | 1.23 (1.03,1.49) | | 1.23 (1.03,1.49) | | 1.23 (1.03,1.49) | 1.23 (1.03,1.49) | | 1.23 (1.03,1.49) | 1.23 (1.03,1.49) | |
| LDLC (mmol/L) | 2.45 (1.94,3.02) | | 2.45 (1.94,3.02) | | 2.45 (1.94,3.02) | 2.45 (1.94,3.02) | | 2.45 (1.94,3.02) | 2.45 (1.94,3.02) | |
| TG (mmol/L) | 0.79 (0.59,1.10) | | 0.79 (0.59,1.10) | | 0.79 (0.59,1.10) | 0.79 (0.59,1.10) | | 0.79 (0.59,1.10) | 0.79 (0.59,1.10) | |
| SOD (IU/mL) | 58.53 (53.43,63.24) | | 58.53 (53.43,63.24) | | 58.53 (53.43,63.24) | 58.53 (53.43,63.24) | | 58.53 (53.43,63.24) | 58.53 (53.43,63.24) | |
| MDA (μmol/L) | 4.71 (3.73,5.79) | | 4.71 (3.73,5.79) | | 4.71 (3.73,5.79) | 4.71 (3.73,5.79) | | 4.71 (3.73,5.79) | 4.71 (3.73,5.79) | |
| WBC (10^9^/L) | 5.30 (4.30,6.40) | | 5.30 (4.30,6.40) | | 5.30 (4.30,6.40) | 5.30 (4.30,6.40) | | 5.30 (4.30,6.40) | 5.30 (4.30,6.40) | |
| HGB (g/L) | 118 (106,131) | | 118 (106,131) | | 118 (106,131) | 118 (106,131) | | 118 (106,131) | 118 (106,131) | |
| Abbreviations: CRP, C reactive protein; ALB, plasma albumin; CHO, total cholesterol; CREA, plasma creatine; HDLC, high-density lipoprotein cholesterol; LDLC, low-density lipoprotein cholesterol; SOD, superoxide dismutase; TG, triglyceride; SOD, superoxide dismutase; MDA, malondialdehyde; WBC, white blood cell count; HGB, hemoglobin.  M (SD), mean (standard deviation); M (IQR), median (interquartile range). | | | | | | | | | | |

| **Supplementary Table S2.** Sensitivity analysis of 25(OH)D (nmol/L) with risk of frailty. | | |
| --- | --- | --- |
| Variables | Model 1 ^a^ | Model 2 ^b, c^ |
| Continuous | 0.982(0.963,0.992) ^***^ | 0.974(0.964,0.983) ^***^ |
| Categories |  |  |
| ≤26.13 | 3.243(2.110,4.963) ^***^ | 3.239(2.113,4.967) ^***^ |
| 26.13–35.89 | 2.348(1.515,3.601) ^***^ | 2.341(1.519,3.609) ^***^ |
| 35.89–50.00 | 1.698(1.082,2.659) ^*^ | 1.703(1.088,2.664) ^*^ |
| >50.00 | reference | reference |
| ^a^ Analysis in older adults with complete variables (N=1107, the number of cases=346).  ^b^ Analysis in older adults with multiple imputation (N=1324, the number of cases=426).  ^c^ *p*-value for Hosmer-Lemeshow test was 0.168; the prediction in accuracy was 80.6% in model 2.  Both models were adjusted for socio-demographics (age, sex, marital status, residence, education level, and co-residence), health characteristics (smoking, drinking, regular exercise, hypertension, diabetes mellitus, heart diseases, cerebrovascular diseases, and respiratory diseases) and confounding biomarkers (CRP, ALB, CHO, CREA, HDLC, LDLC, TG, SOD, MDA, WBC, and HGB), OR (95% CI). | | |
